# Insect live larvae as a new nutritional model in duck: effects on gut health

**DOI:** 10.1186/s42523-024-00316-5

**Published:** 2024-05-29

**Authors:** Elena Colombino, Marta Gariglio, Ilaria Biasato, Ilario Ferrocino, Sara Pozzo, Emma Fragola, Elena Battisti, Stefania Zanet, Ezio Ferroglio, Maria Teresa Capucchio, Achille Schiavone

**Affiliations:** 1 Department of Veterinary Sciences, University of Turin, Grugliasco, 10095 TO Italy; 2 Department of Agricultural, Forestry and Food Sciences, University of Turin, Grugliasco, 10095 TO Italy; 3 National Research Council, Institute of Agricultural Biology and Biotechnology (CNR-IBBA), Milano, 20133 MI Italy; 4 National Research Council, Institute of Science of Food Production, Grugliasco, 10095 TO Italy

**Keywords:** Black soldier fly, Yellow mealworm, *Hermetia illucens*, *Tenebrio molitor*, Microbiota, Volatile fatty acid

## Abstract

**Background:**

This study aimed to evaluate the effects of *Hermetia illucens* (Black soldier fly-BSF) and *Tenebrio molitor* (Yellow mealworm-YMW) live larvae as a new nutritional model on duck’s gut health, considering gut histomorphometry, mucin composition, cytokines transcription levels, and microbiota. A total of 126, 3-days-old, females Muscovy ducks were randomly allotted to three dietary treatments (6 replicates/treatment, 7 birds/pen): (i) C: basal diet; (ii) BSF: C + BSF live larvae; (iii) YMW: C + YMW live larvae. BSF and YMW live larvae were administered on top of the basal diet, based on the 5% of the expected daily feed intake. The live weight, average daily gain, average daily feed intake and feed conversion ratio were evaluated for the whole experimental period. On day 52, 12 ducks/treatment (2 birds/replicate) were slaughtered and samples of duodenum, jejunum, ileum, spleen, liver, thymus and bursa of Fabricius were collected for histomorphometry. Mucin composition was evaluated in the small intestine through histochemical staining while jejunal MUC-2 and cytokines transcription levels were evaluated by rt-qPCR. Cecal microbiota was also analyzed by means of 16 S rRNA gene sequencing.

**Results:**

Birds’ growth performance and histomorphometry were not influenced by diet, with a proximo-distal decreasing gradient from duodenum to ileum (*p* < 0.001), respecting the physiological gut development. Mucin staining intensity and MUC-2 gene expression did not vary among dietary treatments, even though mucin intensity increased from duodenum to ileum, according to normal gut mucus physiology (*p* < 0.001). Regarding local immune response, IL-6 was higher in YMW group when compared to the other groups (*p* = 0.009). Insect live larvae did not affect cecal microbiota diversity, but BSF and YMW groups showed a higher presence of *Helicobacter*, *Elusimicrobium*, and *Succinatimonas* and a lower abundance of Coriobacteriaceae and *Phascolarctobacterium* compared to C birds (*p* < 0.05).

**Conclusions:**

The use of BSF and YMW live larvae as new nutritional model did not impair gut development and mucin composition of Muscovy ducks, but slightly improved the intestinal immune status and the microbiota composition by enhancing regulatory cytokine IL-6 and by increasing minor Operational Taxonomic Units (OTUs) involved in short-chain fatty acids production.

## Background

Duck meat production is rapidly growing worldwide, increasing from 2.9 million tons in 2000 to 7.2 million tons in 2018, with an annual growth rate of 3.2% [1]. China continues to be the leading producer of meat ducks, followed by France, Myanmar, the UK, and the USA [2]. This emerging interest in duck rearing can be due to their potential value as an alternative, sustainable livestock [3]. In fact, ducks have several advantages over other poultry species: they are hardy, they have higher disease resistance, they are easy to manage and they are excellent foragers with the ability to adapt to different feeds [3]. Moreover, genetic improvement programs for meat-type ducks have been successfully carried out to enhance their productive performance [2]. Particularly, the Pekin duck is the predominant meat breed followed by Muscovy and Mule ducks thanks to their faster growth rates, efficient feed conversion, and better meat quality, with higher meat yield and lower fat deposition in comparision to the other duck species [1].

However, this higher demand for duck meat has led to a transformation of the production systems from traditional to large-scale intensive farms, generating several concerns about animals’ well-being [4]. In fact, previous research proved that intensive rearing systems generally led to higher environmental stress compared to free-range rearing system, having a detrimental effect on gut health [5, 6].

Gut health can be defined as the absence, prevention, or avoidance of intestinal disease and it is crucial for the efficient conversion of feed into its basic components for optimal nutrient absorption [7]. Two functional entities are key to achieving and maintaining gut health: the intestinal microbiota and the gut barrier, which encompasses adequate morphometry, proper mucin production, and an effective mucosal immune system [8]. All of these components can be negatively affected by different types of stressors (e.g., heat, excessive amount of feed, overstocking) [9, 10]. Particularly, stress can impair morphology and cause chronic low-grade inflammation in the duck’s gut, compromising digestion, nutrient absorption, and as a consequence bird health, performance, and welfare [5, 6]. For these reasons, research focused on new strategies to maintain duck welfare and gut health in intensive production systems [11, 12].

In this context, *Hermetia illucens* (black soldier fly, BSF) and *Tenebrio molitor* (yellow mealworm, YMW) live larvae have been proved to have dual beneficial effects in poultry. On one hand, insect live larvae can be used as a new nutritional model to increase locomotor activity, litter-directed behaviors, foraging, and to reduce stress and fearfulness [13]. On the other hand, insects administered in small amounts can act as prebiotics thanks to different active coumpounds such as lauric acid, defensins and chitin, which are showing antibacterial, antiviral and immunomodulatory properties [14]. Previous studies are available in poultry, demonstrating that insect live larvae did not impair gut morphology and mucin composition, but positively modulated gut cytokines transcription levels and microbiota [15]. However, to the author’s knowledge, no similar studies are available on Muscovy ducks.

Thus, this study aimed to evaluate the effects of BSF and YMW live larvae as a new nutritional model on duck’s gut health, considering gut morphometry, mucin composition, selected cytokines transcription levels, and microbiota composition.

## Results

### Growth performance

The overall growth performance results are reported in Table 1. The final LW and the overall ADG, ADFI and FCR were not affected by the dietary treatments (*p* > 0.05). Detailed results regarding the growth performance of the birds are reported in Gariglio et al. [16].

**Table 1:** Growth performance of Muscovy ducks fed BSF and YMW live larvae provided at 5% of the expected ADFI [mean (SD)]

Items	Age	Dietary treatments			*p*-value
		C	BSF	YMW	
LW (g)	3 d	80.7 (1.72)	79.9 (3.56)	80.4 (2.45)	0.890
	55 d	2589 (26.4)	2634 (52.1)	2607 (60.1)	0.297
ADG (g/d)	3–55 d	48.2 (0.516)	49.1 (1.03)	48.6 (1.15)	0.294
ADFI^1^ (g/d)	3-55d	113 (5.52)	116 (8.86)	115 (2.78)	0.658
FCR (g/g) + larvae	3–55 d	2.34 (0.114)	2.37 (0.191)	2.34 (0.058)	0.928

C: control; BSF, black soldier fly; YMW: yellow mealworm; LW: live weight; ADG: average daily gain; ADFI: average daily feed intake (on a dry matter basis, including the larvae intake); FCR: feed conversion ratio (on a dry matter basis, including the larvae intake)

^1^ADFI (g/d) + larvae 3-55d (as fed): C = 124.8; BSF = 126.5 + 6.2; YMW = 124.4 + 6.2.

### Histomorphological investigations

Data regarding morphometrical evaluation are reported in Table 2. Non-significant differences were recorded among the dietary treatments for villus height (Vh), crypt depth (Cd), and villus height to crypt depth ratio (Vh/Cd) in the duodenum, jejunum, and ileum (*p* > 0.05). Regardless of diet, Vh and Cd depended on the intestinal segment, showing a proximo-distal decreasing gradient from the duodenum to the ileum (*p* < 0.001).

**Table 2:** Effects of 5% dietary BSF and YMW live larvae supplementation on gut morphology of the Muscovy ducks (*n* = 12/treatment) [mean (SD)]

Item	Diet (D)			Intestinal segment (I)			*p*-value		
	C	BSF	YMW	DU	JE	I	D	I	DxI
Villus height (Vh)	0.79 (0.24)	0.78 (0.23)	0.84 (0.24)	1.03^a^ (0.18)	0.76^b^ (0.23)	0.62^c^ (0.23)	0.147	< 0.001	0.833
Crypt depth (Cd)	0.06 (0.02)	0.07 (0.02)	0.07 (0.02)	0.08^a^ (0.02)	0.06^b^ (0.02)	0.05^c^ (0.02)	0.256	< 0.001	0.880
Vh/Cd	12.2 (3.29)	11.1 (3.29)	12.9 (3.29)	12.3 (3.29)	12.1 (3.29)	11.6 (3.29)	0.063	0.594	0.910

C: control; BSF: black soldier fly; YMW: yellow mealworm; DU: duodenum; JE: jejunum; I: ileum

Table 3 summarized the histopathological findings in the main organs of the ducks. Diet did not influence the severity of the observed histopathological lesions in the liver, thymus, Bursa of Fabricius, and gut (*p* > 0.05). Regardless of diet, the liver showed mild to severe, multifocal to diffuse vacuolar degeneration along with mild and multifocal lymphoplasmacytic inflammation. Bursa of Fabricius and thymus presented from absent to mild follicular depletion and cortical depletion, respectively. An absent to mild and multifocal lymphoplasmacytic enteritis was also recorded in the small intestine.

**Table 3:** Effects of 5% dietary BSF and YMW live larvae supplementation on the main organs of the Muscovy ducks (*n* = 12/treatment)

Item	Diet			*p*-value
	C	BSF	YMW	
Liver				
Degeneration, median (IR)	0.00 (0.0–1.0)	0.50 (0.0-1.5)	0.50 (0.0-1.1)	0.454
Inflammation, median (IR)	0.00 (0.0–0.0)	0.00 (0.0-0.5)	0.00 (0.0-0.1)	0.110
Spleen, mean (SD)	0.03^a^ (0.13)	0.35^b^ (0.45)	0.28^b^ (0.25)	0.025
Thymus, median (IR)	0.00 (0.0-0.5)	0.00 (0.0-0.1)	0.00 (0.0-0.5)	0.438
Bursa of Fabricius, median (IR)	0.50 (0.0-0.6)	0.25 (0.0-0.5)	0.50 (0.0–1.0)	0.306
Gut, median (IR)	0.75 (0.0–1.0)	1.00 (0.0-1.6)	0.00 (0.0–2.0)	0.716

C: control; BSF: black soldier fly; YMW: yellow mealworm; SD: standard deviation; IR: interquartile range

On the contrary, the spleen showed a statistically significant difference among dietary treatments, being the white pulp hyperplasia greater in YMW and BSF groups compared to the control (*p* = 0.025).

### Mucin staining intensity

Table 4 reported the results for the histochemical quantification of mucin in duck’s gut. Non-significant differences were observed for all the evaluated mucins among dietary treatments (*p* > 0.05). However, sialomucins, sulfomucins and total mucins depended on gut segment, showing a proximo distal increasing gradient from duodenum to ileum (*p* = 0.001).

**Table 4:** Mucin histochemical quantification in the small intestine of the Muscovy ducks receiving dietary BSF and YMW live larvae supplementation (*n* = 12/treatment) [mean (SD)]

Item	Diet (D)			Intestinal segment (I)			*p*-value		
	C	BSF	YMW	DU	JE	I	D	I	DxI
Neutral mucins	3.80 (1.48)	3.72 (1.24)	3.63 (1.12)	3.44 (1.19)	3.91 (1.48)	3.81 (1.13)	0.822	0.696	0.126
Sialomucins	3.73 (1.79)	3.61 (1.68)	3.88 (2.37)	2.78 (1.64)	3.98 (1.81)	4.50 (2.03)	0.991	0.001	0.129
Sulfomucins	3.40 (1.77)	3.43 (1.55)	3.41 (1.43)	2.46 (0.92)	3.46 (1.27)	4.35 (1.82)	0.957	0.001	0.154
Total mucins	10.45 (3.55)	10.86 (3.28)	10.81 (3.80)	8.69 (2.50)	10.95 (3.37)	12.51 (3.60)	0.933	0.001	0.171

C: control; BSF: black soldier fly; YMW: yellow mealworm; DU: duodenum; JE: jejunum; I: ileum; SD: standard deviation

### Real-time quantitative PCR (rt-qPCR)

Cytokines and MUC-2 transcription levels in the jejunum of Muscovy ducks are summarised in Table 5. IL-6 transcription levels were influenced by diet, being higher in YMW group when compared to the other groups (*p* = 0.009). The other evaluated cytokines and MUC2 were not influenced by diet (*p* > 0.05).

**Table 5:** Relative mRNA expression of gut cytokines and MUC-2 in jejunal tissue of Muscovy ducks receiving 5% dietary BSF and YMW live larvae supplementation (*n* = 12/treatment) [mean (SD)]

Item	Diet			*p*-value
	C	BSF	YMW	
IL-2^1^	0.872 (0.39)	0.685 (0.36)	0.819 (0.32)	0.716
IL-4^1^	1.309 (1.34)	0.933 (0.87)	1.887 (1.37)	0.348
INF-γ^1^	0.895 (0.42)	0.706 (0.52)	1.103 (0.62)	0.296
TNF-α^1^	1.132 (0.52)	0.891 (0.63)	1.485 (0.66)	0.126
IL-6^1^	1.091^ab^ (0.46)	0.848^a^ (0.54)	1.304^b^ (0.46)	0.009
MUC-2^1^	1.707 (1.46)	0.769 (0.68)	1.786 (1.13)	0.086

C: control; BSF: black soldier fly; YMW: yellow mealworm; IL: interleukin; INF: interferon; MUC: mucin; TNF: tumor necrosis factor; SD: standard deviation

^1^Reference genes (B-actin and GAPDH) were used for normalization of the real-time PCR

### Caecal Microbiota composition and volatilome

**Fig. 1:**
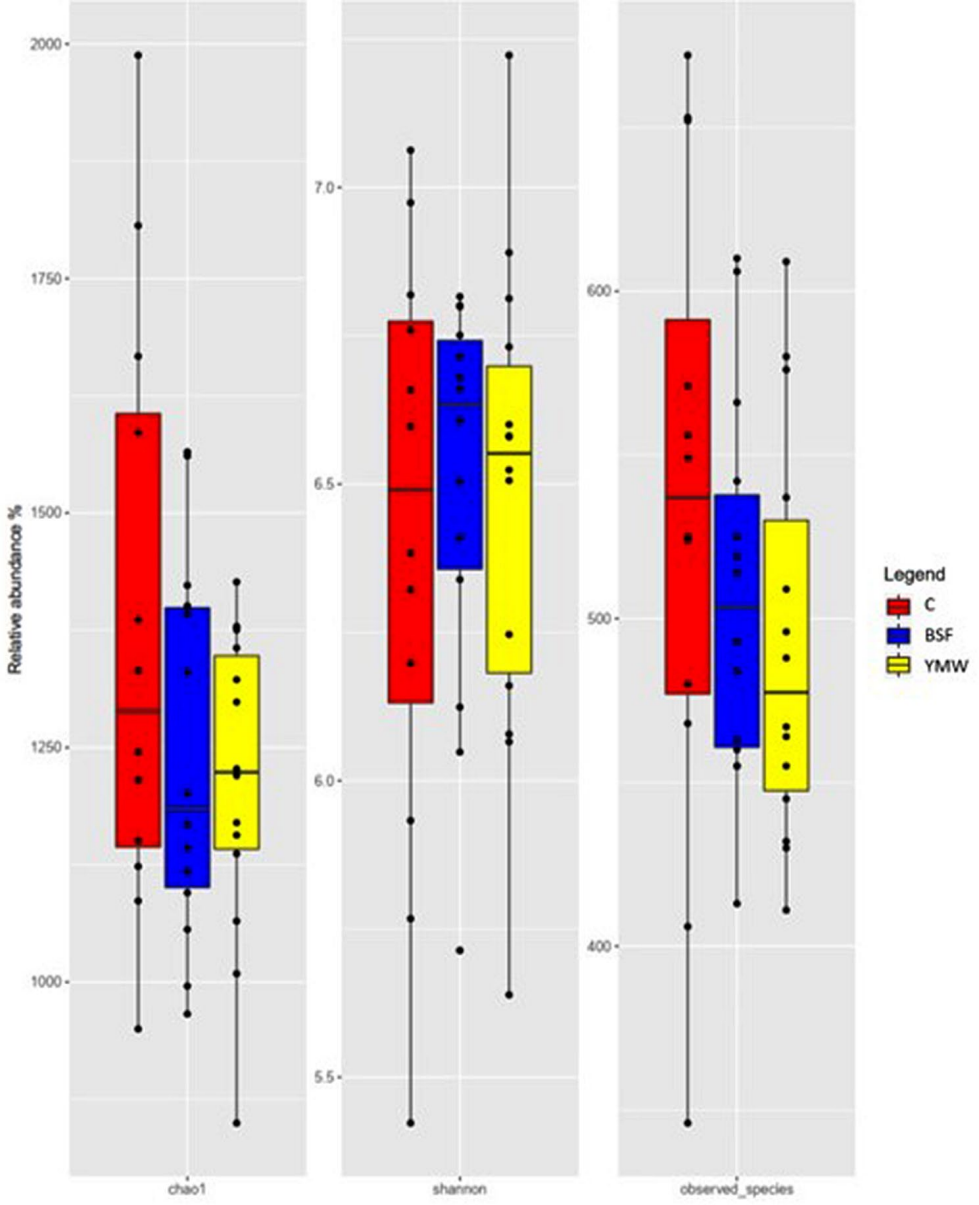
Alpha diversity measures (Chao1, Shannon and observed species indexes) of the ceaca microbiota in the three dietary treatments (C: control; BSF: black soldier fly; YMW: yellow mealworm)

**Fig. 2:**
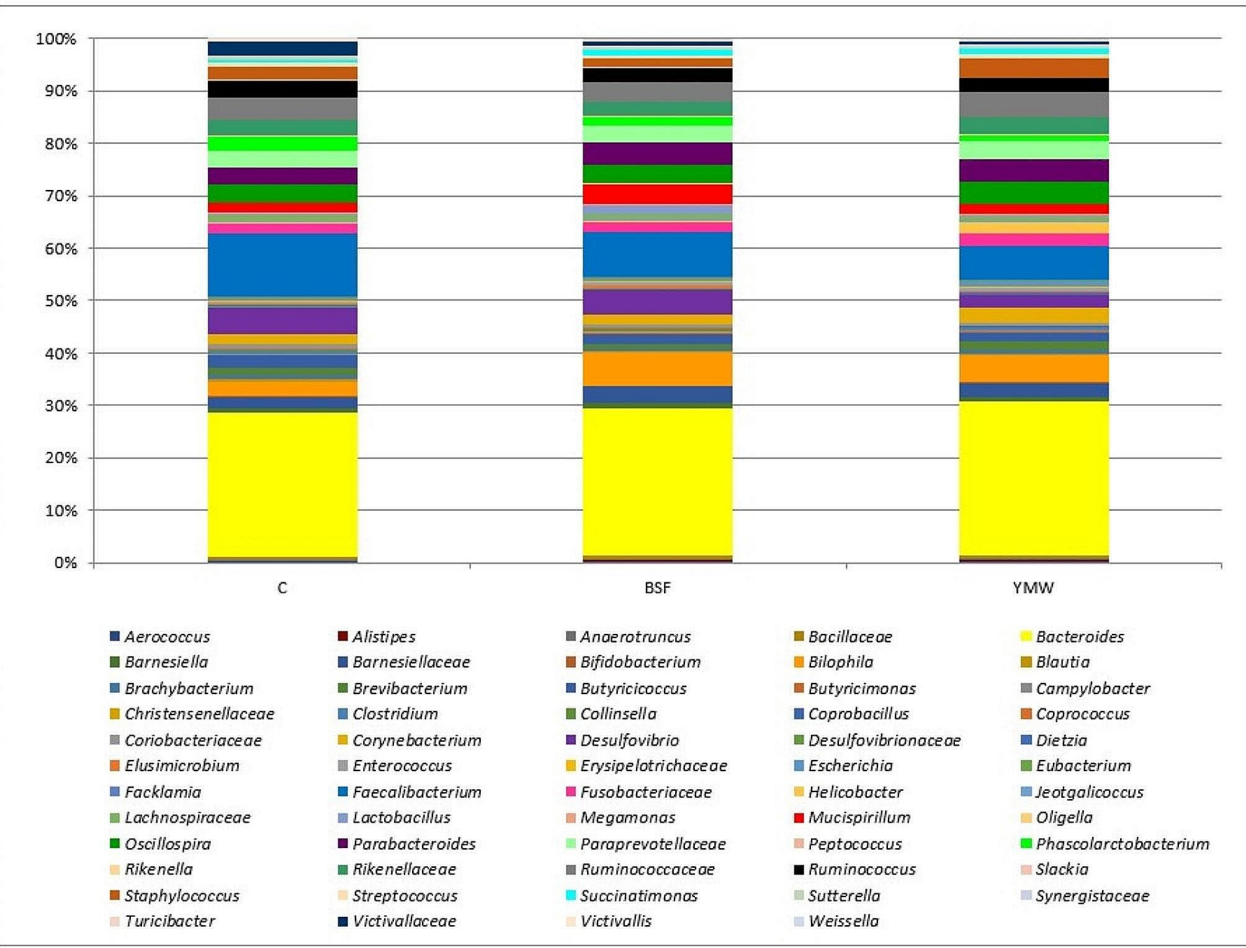
Relative abundance of bacterial taxa in the three dietary treatments (C: control; BSF: black soldier fly; YMW: yellow mealworm)

**Fig. 3:**
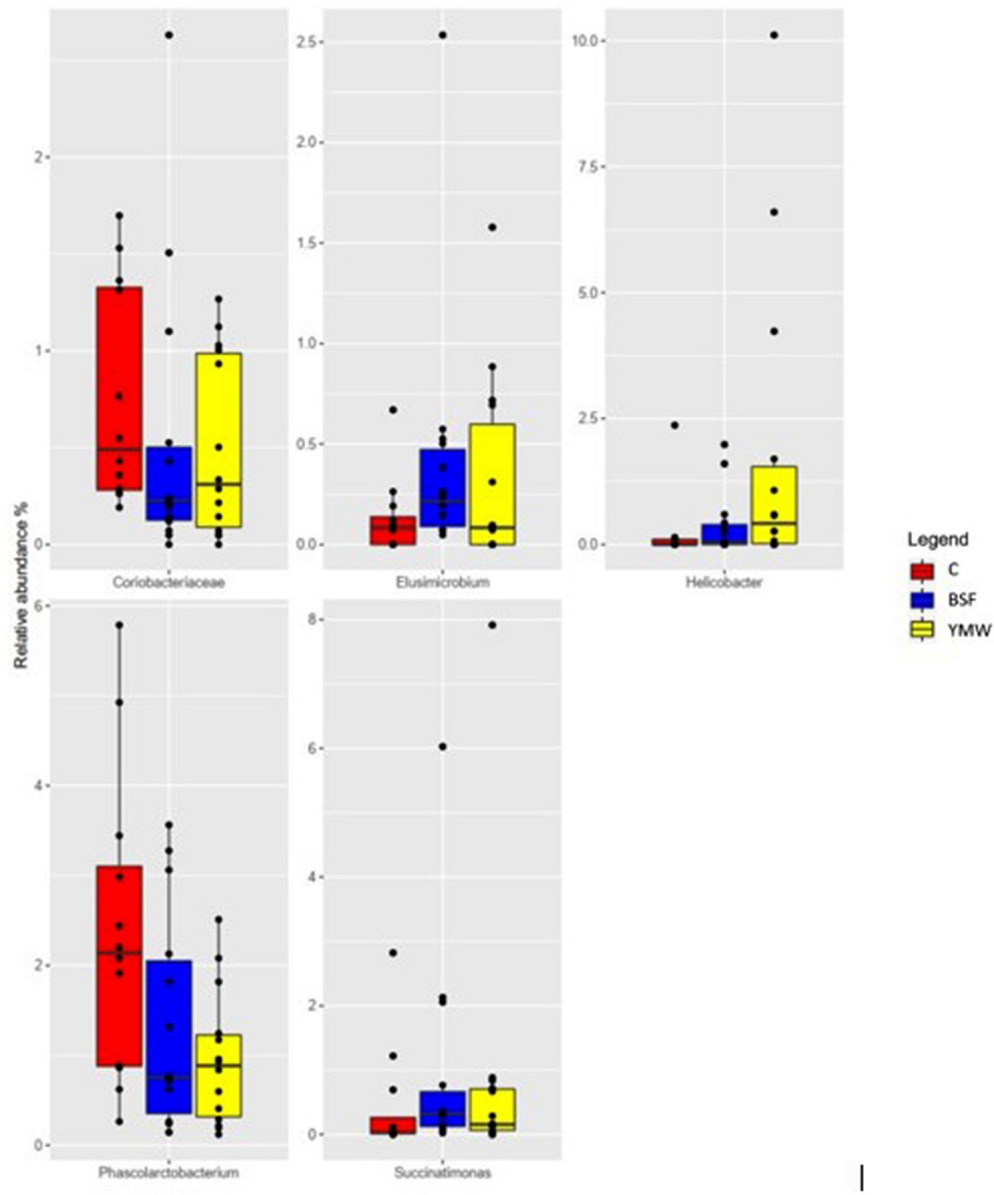
Differentially abundant OTU as a function of the three dietary treatments (C: control; BSF: black soldier fly; YMW: yellow mealworm)

A total of 36 caecal samples were obtained and sequenced. After sequencing and quality filtering, 1,420,947 reads were used for downstream analysis with an average value of 33,832 reads/sample. No significant differences in alpha diversity measures (Shannon and Chao1 indexes) were observed among the three experimental treatments (Fig. 1, *p* > 0.05). In all the three dietary treatments, the microbiota was characterized by the presence of Ruminococcacae and Desulfovibrio family. At genus level, *Bacteroides*, *Faecalibacterium* and *Bilophila* were the most abundant ones (Fig. 2). However, the minor Operational Taxonomic Unit (OTU) fraction (relative abundance < 5%) varied among the dietary treatments (*p* < 0.05, Fig. 3). In particular, BSF and YMW groups showed the highest presence of *Helicobacter, Elusimicrobium*, and *Succinatimonas* and a lower abundance of Coriobacteriaceae and *Phascolarctobacterium* when compared to control (*p* < 0.05, Fig. 3).

Regarding volatilome, non-significant differences were observed for butirric, isobutirric, valeric, isovaleric, propionic and acetic acid as well as for the total amount of aldehydes, ketones, alcohols, acids, and esters among the dietary groups (Table 6, *p* > 0.05).

**Table 6:** Composition of the cecal volatilome of Muscovy ducks receiving 5% dietary BSF and YMW live larvae supplementation (*n* = 6/treatment) [mean (SD)]

Volatile fatty acids	Diet			*p*-value
	C	BSF	YMW	
Acetic acid	5.58 (0.23)	5.15 (0.37)	5.24 (0.21)	0.196
Butirric acid	6.63 (0.17)	6.64 (0.19)	6.59 (0.15)	0.872
Isobutirric acid	5.90 (0.44)	5.55 (0.31)	5.57 (0.34)	0.216
Propionic acid	5.38 (0.16)	5.34 (0.15)	5.42 (0.19)	0.722
Valeric acid	6.19 (0.49)	6.53 (0.22)	6.37 (0.21)	0.243
Isovaleric acid	6.31 (0.15)	6.51 (0.37)	6.47 (0.10)	0.318
Total alcohols^1^	49.9 (1.42)	49.7 (0.95)	50.0 (1.15)	0.895
Total aldehydes^2^	46.8 (2.30)	46.8 (1.28)	46.9 (1.43)	0.980
Total ketones^3^	17.1 (1.52)	18.1 (1.35)	18.6 (1.21)	0.218
Total acids^4^	17.1 (0.73)	17.5 (0.65)	17.2 (0.84)	0.631
Total esters^5^	31.6 (0.59)	31.3 (0.51)	31.6 (0.63)	0.567

C: control; BSF: black soldier fly; YMW: yellow mealworm. ^1^ ethanol; isopropanol; 3-methyl-1-butanol; 1-hexanol; 1-propanol; phenol; 4-methyl phenol; 4-ethyl phenol. ^2^ 2-methyl butanal; 3-methyl butanal; hexanal; propanal; 2-methyl propanal; butanal; benzeneacetaldehyde; benzaldehyde; 2-phenylcrotonaldehyde. ^3^ acetone; 2-butanone; 2,3 butanedione; 3-hydroxy-2-butanone; 3-ottanone. ^4^ 4-methyl pentanoic acid; Hexanoic acid, 2-methyl butanoic acid. ^5^ ethyl propanoate; ethyl acetate; ethyl anteiso valerate; ethyl butanoate; methyl 4,6 dimethyl octanoate; ethyl isovalerate

## Discussion

Insect meals, particularly BSF and YMW meals, have been already tested as innovative protein sources in duck nutrition with promising results as they maintained adequate growth performances and they did not impair gut development and general health of the birds [17, 18]. However, insects have been recently proposed as a new nutritional model for poultry, improving bird’s welfare in intensive farming systems but also acting as prebiotics thanks to their content in chitin, a bioactive compound with antimicrobial and immunostimulant effects [19, 20].

Regarding gut morphometry, non-significant differences were recorded for Vh, Cd and Vh/Cd. These results are in accordance with Gariglio et al. [17] who observed that BSF meal did not impair Muscovy ducks’ gut development. Moreover, Vh and Cd showed a proximo-distally decreasing gradient from the duodenum to the ileum (*p* = 0.001), showing a physiological development of the gut [21, 22]. Thus, intestinal morphological structure and functionality are strictly linked with growth performance and in the present study the final live weight (2594.80±37.11 g) was in accordance with the rearing guide for this specific duck genotype (Canedins R61 Barred blue, Grimaud Freres Selection, France) at the same ages [23], suggesting that insect live larvae have any negative impact on productive performance [7].

In addition, BSF and YMW live larvae did not influence the severity of the histopathological alterations observed in gut, liver, thymus and Bursa of Fabricius, suggesting that they did not have any adverse effects on animal general health. These results are in accordance with previous works using different BSF and YMW inclusion levels in broiler chickens [24, 25] and Muscovy ducks [17]. However, greater white pulp hyperplasia was recorded in the spleen of BSF and YMW groups. This finding is in accordance with Bovera et al. [26] who found higher spleen weight in broiler fed insect meal. This can be attributed to the insect chitin content which lead to an increase in the activity of the immune system, indicating a better disease resistance and immune response of the birds [27]. In fact, it has been previous reported that insect meal can increase the proliferation of CD8 + lymphocytes [28, 29].

The use of insect live larvae as new nutritional model did not impair neutral, acid sialilated, acid sulphated, total mucins or the MUC-2 transcription levels in the jejunum. Previous studies have demonstrated that dietary factors can alter mucin secretion ad as a consequence digesta viscosity, integrity of the mucus layer, and nutrient absorption [30]. The lack of effects observed after the administration of insect live larvae at the dosage used in the present study is in accordance with previous findings in chickens [15, 31]. On the contrary, Biasato et al. [32], and Biasato et al. [33], observed a higher mucin staining intensity in chickens fed 5% of BSF or YMW meal. These heterogeneous results can be due to the different amount of bioactive compounds and to the different form in which insects were provided. In fact, BSF and YMW live larvae had a higher water content (around 70%) and, as a consequence, a lower concentration of nutrients and bioactive compounds compared to BSF and YMW meals which dry matter (DM) amount is over 90% [34]. This hypothesis is supported by the more pronounced effects on mucin composition in chickens fed higher doses of BSF and YMW meals [24, 35]. However, the mechanism through which chitin and the other bioactive compounds can modulate MUC-2 transcription levels and mucin secretion is still unclear [36]. It has been hypothesized that chitin and chitosan could reach the gut and affect intestinal glycosylation, further affecting the MUC2 secretion [37].

Considering intestinal cytokines transcription levels, only IL-6 was significantly higher in YMW group compared to BSF (*p* = 0.009). To date, IL-6 has both proinflammatory and anti-inflammatory properties [38]. On one hand, it is a potent inducer of the acute-phase protein response [39]. On the other, it down-regulates the synthesis of the proinflammatory cytokines, having little effect on the synthesis of anti-inflammatory cytokines. The net result of these immunologic effects place IL-6 among the “regulatory” cytokine group [38]. Many studies have shown that the innate immune response can be modulated by dietary supplementation in broilers [40–42]. Particularly, BSF and YMW live larvae administration as new nutritional model in chickens showed lower IL-2 transcription levels in the jejunum of YMW group [15]. Even if different cytokines were influenced by the use of insect live larvae in chickens and ducks, they seem to drive the intestinal immune status towards an anti-inflammatory pattern by reducing pro-inflammatory IL-2 in chickens and by increasing the IL-6 in ducks. These results are in accordance with Yu et al. [43], who reported the down-regulation of pro-inflammatory cytokines and the up-regulation of the anti-inflammatory ones in pigs fed with BSF meals. The greatest effects observed in the YMW group compared to BSF group could be attributed to the different bioactive compounds content of the two insect species, including chitin. In fact, YMW larvae are reported to be less rich in chitin than BSF and as chitin can be sensed by the innate immune system through specific membrane-bound receptors, YMW seemed to provoke lower stimulation of the inflammatory response [44, 45].

Finally, insect live larvae have no negative effect on gut microbiota diversity as no significant differences were recorded for alpha diversity indices. This result is in agreement with the previous studies conducted by Colombino et al. [15] and Martínez Marín et al. [46]. Regardless of diet, the composition of the ducks’ cecal microbiota was characterized by a high presence of Ruminococcacae and Desulfovibrio family. At genus level *Bacterioides, Faecalibacterium* and *Bilophila* were the most abundant ones. These results are partially in accordance with previous works on Pekin and Muscovy ducks [12, 47, 48]. In fact, it is well known that Ruminococcaceae, Desulfovibrio, *Bacteroides* and *Faecalibacterium* are some of the most representative bacterial genus and family in ducks’ microbiota [47]. Firstly, Ruminococcaceae family and *Faecalibacterium* genus are critical for butyrate production, with positive effects on enterocytes nourishment and on the mucosal barrier functions [49]. Secondly, *Bacteroides* are more abundant in the duck ceca than in any other intestinal segment as they have one of the highest hydrolytic activities among all known genera, being recognized as effective degraders of non-digestible carbohydrates and short-chain fatty acids (SCFAs) producers [50]. The SCFAs are particularly important bacterial fermentation end products, and when they are absorbed into the blood, they can maintain and enhance mucosal growth via direct or indirect mechanisms in the gut, as well influencing metabolism systemically [51]. Thirdly, the presence of Desulfovibrio bacteria could be beneficial for the animals as they consume hydrogen for sulphate reduction, helping in the removal of the free hydrogen produced during anaerobic fermentation [52, 53]. However, it has also been reported that this family is able to degrade intestinal mucin, weakening or damaging the intestinal barrier [54]. In this study, no negative changes in terms of mucins have been recorded, excluding a potential negative influence of this family on mucus layer. On the contrary, scarce data are available regarding *Bilophila* genus, which seems to be a consistent member of the anaerobic colonic microbiota of poultry involved in bile acid metabolism [55].

Furthermore, BSF and YMW live larvae influenced the minor OTUs fraction, being BSF and YMW groups characterized by a higher abundance of *Helicobacter, Elusimicrobium*, and *Succinatimonas* and a lower abundance of Coriobacteriaceae and *Phascolarctobacterium* when compared to control. The role of *Helicobacter* genus in the cecum of avian species is still controversial, as it can stimulate the production of SCFAs, but some species -especially *Helicobacter pylori*-could depress mucin synthesis, determining a worsening of gut health [53, 56, 57]. However, the gut structure and development of BSF and YMW groups of the present experiment were not negatively affected, showing that this increase in *Helicobacter* genus had no negative effects on birds’ performance and welfare. Furthermore, *Succinatimonas* spp. can ferment glucose and other carbohydrates to generate large amounts of SCFAs, especially acetate and succinate that can benefit enterocytes development [58]. Moreover, Coriobacteriaceae and *Elusimicrobium* are normal components of the birds’ gastrointestinal microbiota and their variation has no biological significance [59].

These slight variations of the minor OTUs fractions are in accordance with those recorded in chickens receiving BSF and YMW live larvae as a new nutritional model, particularly the increase of the *Helicobacter* genus [15]. However, the lower nutrients amount (in terms of DM) supplied by the live insects’ larvae prevent major changes in cecal microbiota as those reported in chickens fed a higher amount of BSF and YMW meals [31, 32, 35].

Similarly, non-significant differences were recorded for the cecal volatilome. The volatilome refers to all the volatile metabolites produced by the microbiota in the gut, including SCFAs [60]. The lack of differences in volatilome -especially in SCFAs- in the present study is in contrast with previous findings in insect-fed poultry. In fact, Borrelli et al. [51] observed an increase in total SCFAs - particularly propionate and butyrate- in laying hens fed a diet in which soybean meal has been fully replaced by BSF meal. Also, Addeo et al. [61] reported higher percentage of butyric acid in quails fed 1.4% YMW meal as well as increasing percentages of isobutyrate and valeric acids with increasing dosages of YMW meal, from 1.4 to 5.6%. These changes in SCFAs production can be attributed to the massive differences reported in the microbiota of insect-fed group in terms of alpha and beta diversity, with the increase of chitin degrading genera (e.g., *Alkaliphilus transvaalensis, Flavonifractor plautii, Christensenella minuta*) [51]. It can be hypothesized that the lower concentration of nutrients and chitin provided by insect live larvae in the present study was not sufficient to induce any modification in cecal SCFAs concentration [34], even though a slightly increase in minor OTUs of SCFAs producing bacteria has been herein recorded.

## Conclusions

The use of BSF and YMW live larvae as a new nutritional model in Muscovy ducks (5% of the expected average daily feed intake) did not impair gut development and mucin composition. Moreover, the obtained results in terms of growth performance indicate that the dietary provision of insect live larvae can ensure the suitable growth of Muscovy ducks. The observed spleen’s greater white pulp hyperplasia in insect fed birds suggest that BSF and YMW live larvae could increase the immune response of the animals, making them less susceptible to disease. In additions, in the insect fed birds the intestinal immune status resulted slightly improved by enhanced regulatory cytokine IL-6 and by increasing the microbiota minor OTUs fraction involved in SCFAs production.

Future studies should confirm the positive effects of the inclusion of BSF and YMW larvae in poultry nutrition on immune status response, evaluating if a highest amount of larvae could have more influence on this parameters.

## Materials and methods

### Animals and diets

The experimental protocol (ID: 380,576) was approved by the Ethical Committee of the University of Turin (Italy). A detailed description of the experimental design and duck farming conditions is reported in Gariglio et al. [16], which reports results about animals’ growth performance and welfare. Briefly, a total of 126 3-days-old females Muscovy ducklings (Canedins R61 Barred blue, Grimaud Freres Selection, France) were randomly allotted to three dietary treatments (6 replicates/treatment, 7 birds/pen): (i) C: basal diet (Borello Mangimi s.r.l, Bra, Cuneo, Italy); (ii) BSF: C + BSF live larvae; (iii) YMW: C + YMW live larvae. The basal diet, in crumble form, was based on corn, wheat, soybean meal and soybean oil added with a vitamin-mineral premix and provided by Borello Mangimi s.r.l (Bra, Cuneo, Italy). A 2-feeding phase program was applied: started diet (from 3 to 31 days old; crude protein, CP: 19.3% DM and apparent metabolizable energy corrected for nitrogen, AMEn: 11.29 MJ/kg), and grower-finisher diet (from 32 to 55 days old; CP: 17.9% DM and AMEn: 11.48 MJ/kg). The composition of the diets and their nutrient compositions are reported in Table 7. BSF and YMW live larvae were provided daily [62], on top of the basal diet, at the same time (10.00 am) in a plastic plate (diameter: 30 cm) at the 5% of the expected average daily feed intake (ADFI). The total amount of nutrients consumed by the animals is reported in Gariglio et al. [16]. The live weight (LW), average daily gain (ADG), ADFI, and feed conversion ratio (FCR), adjusted with the amount of larvae consumed, were calculated at the pen level for the overall experimental trial. On day 52 of the trial, 12 ducks/treatment (2 birds/replicate, selected based on the average LW), after a feed withdrawal of 12 h, were slaughtered by electrical stunning and bleeding, according to the standard EU regulations.

**Table 7:** Ingredients (g/kg as fed) and nutrient composition of the basal diets

Ingredients	Starter period (3–31 days)	Grower-finisher period (32–52 days)
Corn meal	418	541
Soybean meal	292	234
Bran	53.4	60.0
Common wheat	150	57.8
Wheat meal	34.4	50.0
Soybean oil	10.0	12.0
Calcium carbonate	15.7	22.9
Dicalcium phosphate	12.3	9.90
Sodium bicarbonate	2.50	2.10
Sodium chloride	2.00	1.90
DL-methionine	2.50	1.80
L-lysine HCl	0.90	1.70
Mineral-vitamin premix^1^	4.00	3.00
Optifos 250 bro^2^	1.00	1.00
Avizyme 1500 × ^3^	1.00	1.00
Total	1000	1000
Analyzed nutrient composition		
Dry Matter (%)	90.9	90.6
Crude Protein (%)	19.3	17.9
Ether Extract (%)	2.51	3.23
Ash (%)	6.50	6.52
Calculated nutrient composition		
AMEn (MJ/kg)	11.3	11.5

AMEn: Apparent metabolizable energy corrected with nitrogen retention

^1^Mineral-vitamin premix: vitamin A (retinyl acetate), 12,500 IU; vitamin D3 (cholecalciferol), 3,500 IU; vitamin E (DL-a-tocopheryl acetate), 40 mg; vitamin K (menadione sodium bisulfite), 2.0 mg biotin, 0.20 mg; thiamine, 2.0 mg; riboflavin, 6.0 mg; pantothenate, 15.21 mg; niacin, 40.0 mg; choline, 750.0 mg pyridoxine, 4.0 mg; folic acid, 0.75 mg; vitamin B12, 0.03 mg; Mn, 70 mg; Zn, 62.15 mg; Fe, 50.0 mg; Cu, 7.0 mg; I, 0.25 mg; Se, 0.25 mg

^2^Optifos 250 bro: Phytase (EC 3.1.3.26) (250 OTU/kg diet), Huvepharma, Sofia, Bulgaria

^3^Avizyme 1500X: Complex of Endo 1-4-Beta- Xylanase (EC 3.2.1.8) (256 U/kg), Subtilisin (Ec 3.4.21.62) (2560 U/kg diet) and Alpha-Amylase (EC3.2.1.1) (1472 U/kg diet), Danisco Animal Nutrition, Marlborough, Wiltshire, UK.

### Histomorphological investigations

At slaughter, samples of the duodenum (loop of the duodenum), jejunum (tract before Meckel’s diverticulum), and ileum (the tract before the ileocolic junction) were excised and flushed with 0.9% saline to remove all the content. Also, samples of the liver, spleen, thymus, and Bursa of Fabricius were collected. All the samples were fixed in a 10% buffered formalin solution for histomorphometry. In particular, the fixed tissues were routinely embedded in paraffin wax blocks, sectioned at 5 μm thickness, mounted on glass slides, and stained with Haematoxylin & Eosin (H&E). One slide per each intestinal segment was examined by light microscopy and each slide was captured with a Nikon DS-Fi1 digital camera coupled to a Zeiss Axiophot microscope using a 2.5× objective lens. The NIS-Elements F software was used for image capturing and morphometric analysis was performed by Image®-Pro Plus software (6.0 version, Media Cybernetics, Maryland, USA). The evaluated morphometric indices were Vh (from the tip of the villus to the crypt), Cd (from the base of the villus to the submucosa), and Vh/Cd [21]. These morphometric analyses were performed on 10 well-oriented and intact villi and 10 crypts chosen from the duodenum, jejunum, and ileum [63].

### Mucin composition

The paraffin-embedded sections of the duodenum, jejunum, and ileum were also submitted to triple histochemical staining to evaluate the three different mucin subtypes according to Colombino et al. [15]. Briefly, Periodic Acid Schiff (PAS) was used for staining neutral mucins, Alcian Blue (AB) pH 2.5 for the acidic sialylated mucins and high iron diamine (HID) for the acidic sulfated mucins.

The mucin staining intensity was evaluated on one slide per histochemical staining for each intestinal segment using the Image®-Pro Plus software and expressed as the percentage of the gut mucosal area (covering both the crypts and the villi) that was positive for the evaluated histochemical staining in accordance with Colombino et al. [15].

### Real-time quantitative PCR (rt-qPCR)

At slaughter, jejunum from 12 birds/treatment was aseptically collected, placed 24 h in RNAlater (Sigma-Aldrich, MO, USA) at 4 °C, and then stored at − 80 ◦C until further analysis. Total RNA was extracted using TRIzol reagent (Invitrogen, Carlsbad, CA, USA) in accordance with manufacturer’s instructions. The RNA quality was quantified by Nanodrop 1000 spectrophotometer (Thermo Fisher Scientific Inc., Wilmington, DE, USA) and the ratio (OD260:OD280) ranged from 1.8 to 2.1. Afterward, 2.0 µg of RNA was reverse transcribed to cDNA using the iScript cDNA Synthesis Kit (Bio-Rad Laboratories, Inc., Hercules, CA, USA) according to manufacturer protocol. rt-qPCR was performed using a 7500 Real-Time PCR system (Applied Biosystems, Waltham, MA) in a 20 µL reaction mixture containing 2 µL cDNA, 10 µL of SYBR Green Supermix kit (Bio-Rad Laboratories, Inc., Hercules, CA, USA) and 0.1 µL of forward and reverse primers (40 mM) of the selected genes (Table 8). The final reaction mixture was placed in a thermal cycler and the following program was carried out: initial incubation at 95 °C for 30 s; 40 cycles of denaturation at 95°◦C for 15 s and annealing/extension at 60 °C for 60 s followed by a melting curve analysis (65–95 °C with 0.5 ◦C increments at 2–5 s/step). The relative standard curve method was performed using β-actin and GAPDH as internal control genes to normalize RNA abundance. Each reaction was run in triplicate. Efficiency curves were performed for each primer set using log10 diluted cDNA to obtain efficiency-corrected relative quantification. Amplification efficiency between 90 and 110% was considered acceptable with a correlation coefficient (R2) of 0.99 [64].

**Table 8:** Oligonucleotide primers used for rt-qPCR of duck cytokines and mucin

Type	RNA Target	Primer sequence	GenBank accession no.
Reference gene	β -actin	F:5’- CAGCCATGTATGTAGCCATCCA − 3’ R:5’- CACCATCACCAGAGTCCATCAC-3’	EF667345.1
	GADPH	F:5’- CTCTGTTCGTGGACCTGACCT-3’ R:5’- CAGCAGCAGCCTTCACTACC-3’	AY436595.1
Target gene	TNF-α	F:5’- GGACAGCCTATGCCAACAA-3’ R:5’- CGATCATCTGGTTACAGGAAGG-5’	EU375296.1
	IL-6	F:5’- CAACGACGATAAGGCAGATGGT-3’ R:5’- GAGGATGAGGTGTGTGGTGATTT-3’	AB191038.1
	INF-γ	F:5’-TGACTACAAGAAGTTCAGAGACCT-3’ R:5’- GACTGGCTCCTTTTCCTTTTG-3’	AJ012254.1
	IL-2	F:5’- TTTACCCTGGGGCTACCTAACTTG-3’ R:5’-AGAACAGACACGTTATCACCCACA-3’	AY193713.1
	IL-4	F:5’- AAAGCCTCCACGGTTGTTT-3’ R:5’- TCACGATGTGCAGCAAGTT-3’	MF346730.1
	MUC-2	F:5’- GGGCGCTCAATTCAACATAAGTA-3’ R:5’- TAAACTGATGGCTTCTTATGCGG-3’	XM_005024513.2

GAPDH: glyceraldehyde-3-phosphate; IFN: interferon; IL: interleukin; MUC: mucin; TNF: tumor necrosis factor; F: forward primer; R: reverse primer

### Caecal microbiota and volatilome

At slaughter, samples of caecal content were collected from 18 birds/treatment (3 birds/replicate) and submitted to DNA extraction and sequencing. The DNA was extracted using a commercial kit (RNeasy Power Microbiome KIT, Qiagen, Italy) following the instructions reported by the manufacturer. One microliters of RNase (Illumina Inc. San Diego. CA) was added to digest RNA in the DNA samples with an incubation of 1 h at 37 °C. The DNA was then quantified using the NanoDrop and standardized at 5 ng/µL. The cecal microbiota was then assessed by sequencing the amplified V3–V4 region of the 16 S rRNA gene through the primers and the PCR conditions previously reported by Colombino et al. [15].

Cecal volatilome was determined on 6 birds/treatment (1 bird/pen) using a Head-Space Solid Phase Micro Extraction module (Combi- Pal automated sampler CTC Analytics, Zwingen, Switzerland) equipped with DVB/CAR/PDMS 50/30 µm fiber (Supelco, Bellefonte, USA) and coupled to a gas chromatograph-mass spectrometer (6890 N/5973 N Agilent Technologies, Inc., Wilmington, DE) adapting the protocol previously reported by Battelli et al. [65]. Briefly, an aliquot of 1 g of ceca content was submitted to the following conditions: equilibrium, 10 min at 50 °C during stirring at 250 rpm; exposition, at 50 °C for 40 min maintaining stirring; desorption at 260 °C for 10 min directly in the injection port of the gas chromatography. The separation was achieved on a polar column (Zebron ZB-WAX plus, 60 m × 0.25 mm × 10.25 μm, Phenomenex, Torrance, CA) under the following gas chromatographic condition: carrier gas helium, in constant flow mode at 1.2 ml/min. Acquisition was performed in electronic impact mode. The mass range used was 39–220 amu. The volatile compounds were identified using the Wiley 7n-1 MS library on Agilent MSD ChemStation® software (Agilent Technologies Inc.). Data were expressed as arbitrary units, as log10 of the peak area of the corresponding selected ion.

### Statistical analysis

Statistical analysis was conducted using R software version 4.0.4 (R Foundation for Statistical Computing, Vienna, Austria; http://www.r-project.org). The Shapiro–Wilk test was used to test the normality of the data distribution. The Levene’s test was used to test variance homogeneity. One-way ANOVA was used to analyze the growth performance data, and the results were expressed as the mean and standard error of the mean (SEM).

Data regarding morphometry and mucin staining intensity were analyzed by a robust two-way ANOVA test (trimmed means method) followed by robust pairwise comparisons using the “walrus” R package. Data regarding histopathological scores and volatile fatty acids were analyzed using a one-way ANOVA test followed by Tukey’s post-hoc test. For rt-qPCR, Microsoft Excel was used to convert the quantification cycle (Cq) values to linear units called relative normalized expression and analyzed in accordance with Taylor et al. [66] and Colombino et al. [15]. Data were described as mean and standard deviation (SD). *p* values < 0.05 were considered statistically significant. Regarding microbiota, FLASH software [67] was used to join the reads while QIIME 1.9.0 software [68] was used for the other step as recently described by Ferrocino et al. [69]. Operational Taxonomic Units (OTUs) were picked at 97% of similarity and taxonomy was assessed by Greengenes16S rRNA gene database v. 2013. OTU table was rarefied at the lowest number of sequences and display the higher taxonomy resolution. Alpha diversity was calculated by the vegan package of R [70]. The diversity indices were further analyzed using the pairwise comparisons using the Wilcoxon rank-sum test to assess differences between the diets. Weighted UniFrac distance matrices and OTU tables were used to perform Adonis and Anosim statistical tests in the R environment. A Generalized Linear Model was used to test the importance of insect administration on the relative abundance of OTU.

## Data Availability

No datasets were generated or analysed during the current study.

## Abbreviations

AB: Alcian Blue
ADFI: Average daily feed intake
ADG: Average daily gain
AMEn: Apparent metabolizable energy corrected for nitrogen
BSF: Black soldier fly
C: Basal diet
Cd: Crypt depth
CP: Crude protein
Cq: Quantification cycle
DM: Dry matter
DU: Duodenum
F: Forward primer
FCR: And feed conversion ratio
GAPDH: Glyceraldehyde-3-phosphate
H&E: Haematoxylin & Eosin
HID: High iron diamine
I: Ileum
IFN: Interferon
IL: Interleukin
IR: Interquartile range
JE: Jejunum
LW: Live weight
MUC: Mucin
OTU: Operational Taxonomic Unit
PAS: Periodic Acid Schiff
R: Reverse primer
SCFAs: Short-chain fatty acids
SD: Standard deviation
SEM: Standard error of the mean
TNF: Tumor necrosis factor
Vh/Cd: Villus height to crypt depth ratio
Vh: Villus height
YMW: Yellow mealworm
